# Dynamic body-weight support to boost rehabilitation outcomes in patients with non-traumatic spinal cord injury: an observational study

**DOI:** 10.1186/s12984-020-00791-2

**Published:** 2020-11-30

**Authors:** Justin P. Huber, Lumy Sawaki

**Affiliations:** 1 Department of Physical Medicine and Rehabilitation, University of Kentucky, 2050 Versailles Road, Lexington, KY 40504 USA; 2 Department of Mechanical Engineering, University of Kentucky, 2050 Versailles Road, Lexington, KY 40504 USA; 3 Department of Neurology, University of Kentucky, 2050 Versailles Road, Lexington, KY 40504 USA

**Keywords:** Locomotion, Neuroplasticity, Functional independence measure, Therapeutic technology

## Abstract

**Background:**

Dynamic body-weight support (DBWS) may play an important role in rehabilitation outcomes, but the potential benefit among disease-specific populations is unclear. In this study, we hypothesize that overground therapy with DBWS during inpatient rehabilitation yields greater functional improvement than standard-of-care in adults with non-traumatic spinal cord injury (NT-SCI).

**Methods:**

This retrospective cohort study included individuals diagnosed with NT-SCI and undergoing inpatient rehabilitation. All participants were recruited at a freestanding inpatient rehabilitation hospital. Individuals who trained with DBWS for at least three sessions were allocated to the experimental group. Participants in the historical control group received standard-of-care (i.e., no DBWS). The primary outcome was change in the Functional Independence Measure scores (FIM_gain_).

**Results:**

During an inpatient rehabilitation course, participants in the experimental group (n = 11), achieved a mean (SD) FIM_gain_ of 48 (11) points. For the historical control group (n = 11), participants achieved a mean (SD) FIM_gain_ of 36 (12) points. From admission to discharge, both groups demonstrated a statistically significant FIM_gain_. Between groups analysis revealed no significant difference in FIM_gain_ (p = 0.022; 95% CI 2.0–22) after a post hoc correction for multiple comparisons. In a secondary subscore analysis, the experimental group achieved significantly higher gains in sphincter control (p = 0.011: 95% CI 0.83–5.72) with a large effect size (Cohen’s *d* 1.19). Locomotion subscores were not significantly different (p = 0.026; 95% CI 0.37–5.3) nor were the remaining subscores in self-care, mobility, cognition, and social cognition.

**Conclusions:**

This is the first study to explore the impact of overground therapy with DBWS on inpatient rehabilitation outcomes for persons with NT-SCI. Overground therapy with DBWS appears to significantly improve functional gains in sphincter control compared to the standard-of-care. Gains achieved in locomotion, mobility, cognition, and social cognition did not meet significance. Findings from the present study will benefit from future large prospective and randomized studies.

## Introduction

Global greying is a profound, ongoing phenomenon [1]. It refers to the disproportionate increase in our aged population. Studies suggest this global greying is contributing to increased incidence of non-traumatic spinal cord injury (NT-SCI), which may soon surpass that of traumatic spinal cord injury [2]. Evidence from a large US academic healthcare system suggests that a majority of SCI is due to non-trauma [3]. Studies outside the US also suggest the incidence of NT-SCI is significantly greater than its traumatic counterpart. Based on national databases in Australia and national rehabilitation registries in Canada, the ratio of NT-SCI to traumatic SCI approaches 1.8 [2, 4]. Recognizing this shift toward non-traumatic etiology of SCI is important because studies show rehabilitation potential is correlated to the etiology of SCI [5]. Non-traumatic spinal cord injury comprises multiple causes including tumor, inflammatory conditions, vascular diseases, degenerative disc diseases, and intravenous drug-use [2, 4, 6–9]. Although individuals with NT-SCI at inpatient rehabilitation often have better function at admission versus individuals with traumatic SCI, functional outcomes are similar for both groups [10]. This has been attributed to the advanced age of persons with NT-SCI [4, 9, 11]. For individuals with NT-SCI who discharge with persistent functional impairment, they are at significant risk for depression, cognitive dysfunction, and compromised quality of life [12].

After injury to the central nervous system, extensive evidence supports the benefit of intensive, highly repetitive training that is progressively challenging and task-oriented [13–15]. Persons with NT-SCI are often older and have lower neuromuscular reserve compared to the traumatic SCI population [4, 9, 11]. A progressive gait training program then requires an appropriate starting line—the person’s neuromuscular system must be offloaded [16, 17]. In addition, the individual’s fear of falling needs to be managed. This fear might otherwise distract the individual and disrupt therapist efforts to create an intensive, challenging experience. These issues could be addressed using technology. Indeed, with continued pressure by insurance payers to ration human resources and reduce hospital lengths of stay, new technologies will be paramount.

Various technologies have been proposed to address the prior issues, particularly with regards to locomotor training. Strategies described in literature include parallel bars with bracing, static body-weight support (BWS) with overground training, BWS treadmill training (BWSTT), and robot-assisted treadmill training. Among these approaches, studies have shown no clear difference in effect [15, 18, 19]. However, with the continued evolution of technology, new devices are emerging, one of which is dynamic body-weight support (DBWS) for overground therapy.

**Fig. 1:**
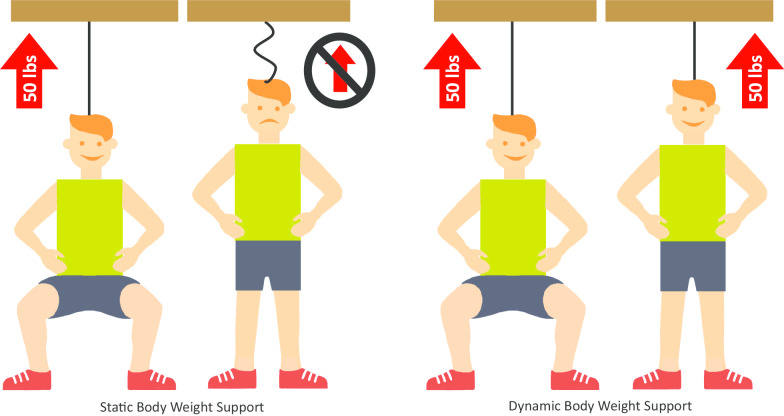
Conceptual difference between static and dynamic body-weight support. Dynamic body-weight support technology continuously adjusts the suspension force using sensors, actuators, and a computer to create a feedback control system. The intent of this system is to create more consistent unloading forces during a participant’s motion

The first BWS systems were appealing to the therapist community due to the added safety provided to both patient and provider. With an expanding, aging patient population, such tools are needed to maximize efforts of a limited workforce. Early BWS systems comprised a simple harness suspension, and they were designed to offload a percentage of a person’s body-weight as measured at rest, that is statically. Hence these systems have been termed static BWS. Recent systems are now designed to both offload body-weight and account for the dynamic forces that occur when a person moves; hence, these systems have been coined dynamic BWS systems (DBWS). Figure 1 illustrates the conceptual difference between static and dynamic BWS systems.

Investigators have begun to explore the impact of DBWS when applied to various populations. For example, Fenuta et al. and Awai et al. explored the effects of DBWS systems on muscle activation patterns and kinematics in healthy participants [16, 20]. A study by Anggelis et al. involved participants with traumatic brain injury participating in therapies with aid of a DBWS system [21]. Within spinal cord injury research, a study by Easthope et al. investigated the effects of DBWS on gait kinematics in participants with chronic incomplete injuries [22]. An additional study by Fenuta et al. explored metabolic demand and muscle activation patterns associated with DBWS use by participants with chronic and incomplete SCI [23]. To our knowledge, the current study is the first to explore the impact of a DBWS system on functional outcome measures in persons with NT-SCI undergoing inpatient rehabilitation.

## Methods

This was a retrospective cohort chart review of individuals discharged from a freestanding inpatient rehabilitation hospital between July 2017 and April 2018. Eleven individuals with diagnosed NT-SCI underwent overground therapy with the DBWS system (ZeroG v3, Aretech LLC, Ashburn, Virginia, USA). The ZeroG device is a cable suspension system that comprises two integrated control systems. To account for vertical forces, a custom series elastic actuator is mounted to an overhead trolley, and this actuator controls rope tension based on input from a force sensor and high resolution linear encoder. To account for horizontal forces (e.g. parallel to trolley track), the trolley itself is instrumented with a DC brushless motor with drive wheel, which controls trolley position based on rope angle measured by a precision potentiometer [24].

Participants in the DBWS group of this retrospective study met specific criteria. The inclusionary criteria were history of NT-SCI (confirmed by medical history and radiographic evidence), satisfaction of inpatient rehabilitation admission criteria (require 3-h therapy daily, require multiple therapy disciplines, require interdisciplinary care including daily physician oversight), and participation in at least three separate therapy sessions with DBWS during the hospital admission. The exclusionary criteria were decided by clinicians and included unstable cardiopulmonary conditions, contractures in the lower extremity, uncontrolled diabetes, severe osteoporosis, severe spasticity, skin lesions that interfere application of DBWS system, and severe syncopal symptoms (lightheadedness or faintness when sitting and/or standing). For the historical control group, eleven individuals discharged from the same facility between March 2017 and May 2017 (prior to the installation of DBWS) were selected based on satisfaction of inclusion and exclusion criteria with the key exception being absence of DBWS. The University of Kentucky Institutional Review Board approved the protocol for this retrospective study, and a waiver of informed consent was secured.

Regarding the use of DBWS, only physical therapists who had been trained and demonstrated competency with the device were involved in its administration. During therapy sessions with the DBWS group and historical control group, participants engaged in activities such as walking, turning, and sit-to-stand transfers. An assortment of traditional tools complimented therapies and included bedside commodes, shopping carts, and stairs. With exception of body-weight support via the ZeroG system, the same exercises and therapy interventions were available to both treatment groups during their rehabilitation course. Sessions involving overground therapy with DBWS were integrated within the standardized 3-h of daily therapy allotted for inpatient rehabilitation facilities per guidelines by the Centers for Medicare and Medicaid Services [25]. This total daily allotment of therapy was the same for both control and experimental groups. That is, no additional physical therapy time was provided to participants in the DBWS group as compared to the historical group. The duration of DBWS use during the 3-h allotted time was ultimately per discretion of the physical therapy team and per tolerance of the participant. Of note, while the historical control group received no DBWS via the ZeroG apparatus, patients in this group did have access to alternative forms for BWS including walkers, parallel bars, and bracing.

Primary outcome measures were based on the Functional Independence Measure (FIM) instrument—a scale with proven validity and reliability widely used among rehabilitation facilities [26, 27]. This instrument assesses 18 different functional areas including 13 motor function areas and 5 cognitive function areas. Within each area, persons are assessed and assigned an ordinal value from 1 to 7 with low scores signifying lack of independence and higher scores signifying increasing independence. The summation of the scores for all 18 areas results in a single FIM score (max score of 126). By assessing the FIM score for individuals at admission versus discharge, the absolute difference (FIM_gain_) provides an indicator of the individual’s response to an inpatient rehabilitation course. Furthermore, the efficiency of the person’s functional recovery (FIM_efficiency_) can be estimated by further dividing by hospital duration in days.

Using statistics software (IBM SPSS version 25), an analysis within groups was performed using a paired t-test to determine if discharge FIM scores were significantly different compared to admission scores for each group. Subsequently, an independent t-test was applied to compare differences in the primary outcome measures (FIM_gain_ and FIM_efficiency_) between groups receiving DBWS and receiving standard of care. All statistical tests were two-tailed. For independent t-test, significance was determined against a Bonferroni-adjusted alpha level of 0.0167 to account for multiple comparisons. For statistically significant differences, the Cohen’s *d* statistic was used to describe the effect size. In addition to the primary outcomes, a secondary analysis was performed comparing differences between groups for the FIM subscores (self care, sphincter control, mobility, locomotion, cognition, and social cognition).

## Results

This study included 22 individuals admitted to an inpatient rehabilitation hospital and discharged during the period from March 2017 to April 2018. The mean (SD) age of these participants was 56 (18) years for the DBWS group and 58 (16) for the historical controls. The mean weight of these participants was 165 (37) pounds for the DBWS group and 264 (72) pounds for the historical controls, which was significantly different (p = 0.001; 95% CI 48.4–149.9). The mean length of stay was 21 (12) for the DBWS group and 29 (31) for the historical control group. For both the historical controls and the experimental group, degenerative spine disease was a prominent etiology of non-traumatic spinal cord injury (64% in each group). Table 1 provides a more detailed comparison of the demographic variables and the admission FIM scores for participants in the DBWS group and the historical control group (e.g. standard of care). To analyze for significant differences in group demographics, an independent t-test was applied for continuous variables and chi square tests were applied for categorical variables.

**Table 1:** Participant demographics

Variables	Historical control group	DBWS group
Age (years)		
Mean (SD)	56 (18)	58 (16)
Gender		
Male (%)	64	55
Female (%)	36	45
Weight (lbs)		
Mean (SD)	264 (71.6)^a^	165 (37.0)^a^
BMI		
Mean (SD)	37.4 (8.0)^a^	27.0 (8.6)^a^
Level of injury		
Cervical (%)	18	64
Thoracic (%)	45^a^	0^a^
Lumbar (%)	36	36
Etiology		
Degenerative spine disorders	7	7
Epidural abscess	3	1
Other (Osteomyelitis, Hemorrhage, Inflammation, Tumor)	1	3
FIM at Admission		
Mean (SD)	54 (13)	56 (7)

*DBWS* Dynamic Body-weight Support, *BMI* body mass index, *FIM* Functional Independence Measure

^a^Statistically significant difference

**Fig. 2:**
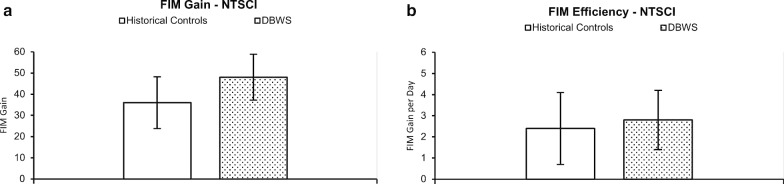
Between groups comparison of functional outcomes. **a** Based on overall gains in the Functional Independence Measure (FIM_gain_) depicted in the graph, participants with non-traumatic SCI (NT-SCI) using dynamic body-weight support (DBWS) achieved similar recovery compared to historical controls treated without DBWS. **b** Regarding the efficiency of the functional gains (FIM_efficiency_), the difference between groups was not significant (right)

With regards to FIM scores, persons with NT-SCI who underwent DBWS gait training achieved a mean (SD) FIM_gain_ of 48 (11) points. Although this appeared higher compared to the historical group receiving standard of care only with mean (SD) FIM_gain_ of 35 (12) points (p = 0.022; 95% CI 2.0–22), the difference failed to reach significance after correcting for multiple comparisons. With regards to FIM_efficiency_ scores, there was no significant difference between the DBWS group and the historical control group receiving standard of care (p = 0.543; 95% CI − 0.97–1.8). Of note, a between group comparison of length of stay showed no significant difference (p = 0.457; 95% CI − 13.8–13.1). Figure 2 provides a graphical depiction comparing these primary outcomes.

**Fig. 3:**
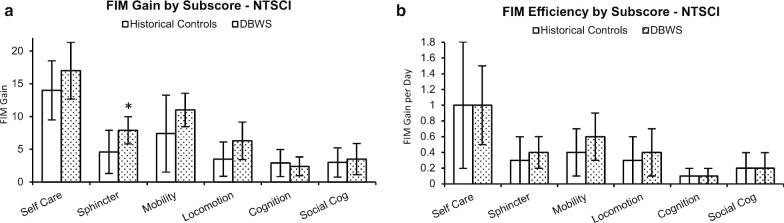
Comparison of functional outcomes based on subscore analysis. **a** Based on analysis of FIM subscores, participants using dynamic body-weight support (DBWS) achieved higher functional gains in the domain of sphincter control with statistical significance denoted by an asterisk. **b** For each domain in the subscores, the analysis revealed no significant differences in the efficiency of functional gains

In a subsequent FIM subscore analysis, the participants with NT-SCI receiving DBWS achieved significantly higher gains in sphincter control with a mean (SD) gain of 7.9 FIM points (p = 0.011; 95% CI 0.83–5.7). While there was a trend for greater gains in locomotion for the DBWS group, this trend did not reach statistical significance (p = 0.026; 95% CI 0.37–5.3) after correcting for multiple comparisons. Self care, mobility, cognition and social cognition domains showed no significant difference. Table 2 provides a summary overview of primary outcomes and subscores for the DBWS group and the historical control group. Figure 3 provides a graphical depiction comparing this subscore analysis between groups.

**Table 2:** Primary outcomes and subscores

Variables	Historical control group	DBWS group
	Mean (SD)	Mean (SD)
FIM gain		
Total	36 (12)	48 (11)
Self care	14 (4.5)	17 (4.3)
Sphincter	4.6 (3.3)^a^	7.9 (2.1)^a^
Mobility	7.4 (5.9)	11 (2.6)
Locomotion	3.5 (2.6)	6.3 (2.9)
Cognition	2.9 (2.1)	2.4 (1.4)
Social cognition	3.0 (2.2)	3.5 (2.4)
Length of stay (days)	29 (31)	21 (12)

Variables	Historical control group	DBWS group
	[range 5–109 days]	[range 10–50 days]
FIM efficiency (points/day)		
Total	2.4 (1.7)	2.8 (1.4)
Self care	1.0 (0.8)	1.0 (0.5)
Sphincter	0.3 (0.3)	0.4 (0.2)
Mobility	0.4 (0.3)	0.6 (0.3)
Locomotion	0.3 (0.3)	0.4 (0.3)
Cognition	0.1 (0.1)	0.1 (0.1)
Social cognition	0.2 (0.2)	0.2 (0.2)

*DBWS* Dynamic Body-weight Support, *FIM* Functional Independence Measure

^a^Statistically significant difference

## Discussion

The present study suggests a potential benefit of using DBWS in overground therapy to improve functional outcomes for individuals with NT-SCI in an acute inpatient rehabilitation setting. More specifically, there was a significant gain in sphincter control in the DBWS group compared to the control group. Improvement in bladder and bowel control is considered high priority by individuals with SCI [28]. While the results of our study require substantiation by larger studies, this initial evidence cannot be understated. The underlying mechanism for improved sphincter control may be explained by shared neural pathways. Prior animal studies suggest overlapping lumbosacral spinal circuitry [29]. It is conceivable that therapies targeting locomotor neurorehabilitation might also be beneficial to bladder and bowel function as shown in recent human studies [30].

With regards to locomotion, a clear trend for higher gains in this subscore is demonstrated in the DBWS group compare to the historical control group. The lack of a statistically significant difference is potentially due to the small sample size of our study and needs to be further investigated. Of note, during the course of this study, no adverse events occurred which supports the safety of a DBWS system within an inpatient setting.

It is important to acknowledge the multiple variables at play in our retrospective study design. For instance, the groups have discrepancies in lengths of stay, which could equate to discrepancies in the duration of therapy administered. However, in our study, the differences in length of stay did not reach statistical significance. Regardless, the duration of DBWS needs to be better controlled in future studies. A typical day of inpatient rehabilitation involves physical therapy administered in a single session or multiple sessions, and each session ranges from 30 to 60 min. The daily duration of DBWS may then be roughly estimated as summation of 30-min intervals minus time needed for participant preparation. By prospectively defining the duration of DBWS or by facilitating more detailed documentation from physical therapists, confidence in study findings will be improved.

Demographics for the historical and DBWS groups revealed some key differences, namely participant weight and level of spinal cord injury. The weight discrepancy between the groups was statistically significant. Conceivably, the increased weight of the historical control group could have negatively contributed to their outcomes. Conversely, the historical control group included significantly more thoracic-level injuries and relatively few cervical-level injuries. This predominance of lower level injuries in the historical control group could have positively contributed to their outcomes. Ultimately, the retrospective nature of this study prevented well-defined durations of DBWS, control of therapy intensity, and matching of groups. These would be important details to address in future prospective clinical studies.

From a technology perspective, better understanding is needed as to how features of DBWS might be favorable or unfavorable to neuroplasticity. Neuroplasticity refers to the adaptive change in neural connections which occur after injury to the central nervous system, and these changes can occur spontaneously or potentially be induced [15, 31]. To achieve the latter, literature highlights the importance of intensive, highly repetitive training with progressive challenge and salience (e.g., task-oriented therapy, functional tasks) [32, 33]. If features of DBWS are favorable in this context, then prioritizing and developing these features will help to advance the technology. We suggest two promising features of DBWS as areas for further research: perceived safety and dynamic performance.

Perceived safety may be beneficial to neuroplasticity in several ways. Patient focus on therapy may be enhanced if risk is reduced, e.g. fall mitigation. Additionally, safety perceived by the physical therapists could increase their willingness to challenge the patient—to set more difficult goals rather than resorting to easier, safer goals. Lastly, repetition in overground therapies might improve. If fatigue-induced failure is no longer associated with a fatigue-induced fall, then a patient might voluntarily attempt higher task repetitions. To explore these speculations in future DBWS research, questionnaire-based assessments of perceived safety within control and intervention groups would be valuable.

Dynamic performance, in this discussion, refers to the transparency of DBWS during unloading of a moving participant. Ideally, a DBWS system would support a patient without causing aberrations to the movement quality (or aberrant perceptions to the patient) as compared to the unsupported patient. A high degree of dynamic performance may be beneficial to neuroplasticity by preserving sameness of the task-oriented therapy. For example, a patient’s experience pushing a rolling walker in the therapy gym with DBWS will feel nearly identical to pushing a rolling walker in the community—not as if walking with cable in tow. Moreover, if dynamic performance can be maintained across a broad spectrum of unloading (e.g. 5% bodyweight support versus 50% bodyweight support), then therapy challenge can be finely tuned and progressed in a more gradual manner without sacrificing task fidelity.

Exceptional research continues to explore this concept of dynamic performance in DBWS systems. One approach has been to measure consistency of vertical unloading force during gait. For example, in a study of participants ambulating fifty feet in the ZeroG DBWS system, a desired 10-lb unloading force demonstrated a root-mean-square error of 0.41-lb while a 120-lb unloading force demonstrated a 1.86-lb root-mean-square error [24]. Another approach is to explore kinematic changes in participants as unloading increases with a DBWS system. In a recent study with healthy participants negotiating stairs, a DBWS system minimally impacted kinematics when unloading was kept below 30% body-weight [34]. These investigations are crucial as DBWS technology is implemented in new forms (e.g. pneumatic actuation, machine learning controls).

### BWS classification: a challenge to assessing impact

Interpreting literature on BWS systems is complicated due to the variety and growing complexity of these systems. Teasing out the impact of dynamic BWS systems requires, first, an appreciation for the dynamic forces at play. Consider a 100 lb person that stands from a seated position. While sitting, a 100 lb antigravity force is applied by the chair seat. When this 100 lb person stands from sitting, a force > 100 lb is generated in order to accelerate upward, and after a subsequent deceleration, this person maintains the static standing position by exerting exactly a 100 lb force. During the course of this person’s transfer, the magnitude of force has continuously changed.

Previous literature has defined dynamic BWS as a system involving force-generating actuators, vertical position and/or force sensors, and controllers. Using feedback control algorithms, output from the actuators is adjusted real-time to minimize errors in the desired position and/or force [35]. In context of prior example, if a 100 lb person is sitting or standing (or transitioning between), the system can actively adjust force magnitude. Thus, the percentage of BWS is maintained during both static and dynamic states.

With this definition in mind, a dynamic BWS system can be distinguished from other BWS strategies, such as static, passive or active systems [35]. For instance, in the study by Sousa et al., patients are supported via an electric cable winch coupled to a load cell [17]. This setup achieves two out of the three elements of a dynamic system, but as a control mechanism is absent, this device is best defined as active BWS. In another study by Franz et al., an active BWS system synchronizes the unloading force to a specific interval of the gait cycle [36]. This setup implements all three elements but a subtle detail is missing. While the unloading force is adjusted during the activity, it is done so in a binary manner (i.e. on/off) and thus does not achieve the continuous variation required for dynamic BWS.

### The Downside of Static BWS

Static BWS systems do not account for the dynamic forces of a moving person. As a result, the percentage of supported body-weight can be irregular or even non-existent during an exercise. This irregularity is undesirable. Studies have shown that static BWS yields non-physiologic ground reaction forces through a person’s feet [35]. This aberration in forces translates to aberrations in sensory afferent information perceived through the feet. With aberrant, non-physiologic afferent feedback, the pattern of leg muscle activation during human locomotion may become less functional [37]. A static BWS system has been shown to adversely affect kinematics including reduced hip range of motion and shortened stride length [36]. In contrast, DBWS can accommodate movement by utilizing sensors, actuators, and computers to adjust rope tension real-time according to the person’s motion. As a result, the individual experiences more consistent unloading and more normalized sensory feedback during therapeutic activity, e.g. locomotor training.

### Dynamic BWS: a step toward realizing the potential of BWS

Like many technologies, BWS systems seek to bridge the gap between limited human resource and the rehabilitation needs of an expanding, aging population. For persons with NT-SCI, a low-technology solution to gait training is parallel bars and the help of multiple therapists. The outcome from this approach might be comparable to current high-technology solutions [15, 18, 19]. However, this low-technology solution places stress on a limited therapist workforce. The incorporation of tools such as BWS systems may reduce the physical burden on therapists while maintaining patient safety [15, 18].

Compared to static systems, a dynamic system may further enhance the safety potential of BWS. For example, static BWS provides crude protection against falls by means of a slack rope becoming suddenly taut. In contrast, dynamic BWS applies a gentler, more gradual tension to a falling patient—a benefit of the onboard computer controller. Furthermore, DBWS uses an overhead carriage to maintain the electrified, force-generating actuators above and away from the patient. In contrast, competing technologies (e.g. treadmills, robotic exoskeletons) often feature actuators in close proximity to the patient. By reducing safety hazards, DBWS permits a more challenging therapy environment, and more challenge will enhance learning [38].

### Opportunities for future investigation

This exploratory study highlights the potential of DBWS and underscores opportunities for future work. The small sample size was a limitation. The retrospective design prevented randomization, allowed variance in intervention parameters, and hampered well-matched groups. That said, the suggested benefit in sphincter control and the promising trend in locomotion are inspiration for future large, prospective, randomized studies.

If clinical findings and trends in our study persist in large prospective studies, then several important questions invite investigation. Foremost, if sphincter control benefits from overground therapy with DBWS, then more sophisticated bowel and bladder outcome measures are needed such as validated assessment scales and urodynamic studies [39]. Also a dose–response analysis will help determine the optimum volume of overground therapy to administer with DBWS and identify the point of diminishing returns. Investigations on subgroups within NT-SCI population will be beneficial. Prior research supports a correlation between cause of NT-SCI and rehabilitation outcome; for example, studies have suggested improved rehabilitation outcomes in NT-SCI secondary to vertebral column degenerative disorders as compared to vascular and infection-related NT-SCI [5]. By studying the response of these subgroups to DBWS therapy, knowledge on high-responders and low-responders would guide allocation of DBWS. Lastly, animal studies of spinal cord injury suggest daily repetitions on the order of thousands are needed for locomotion improvement [40]. Observations from inpatient and outpatient facilities in North America suggest considerably fewer repetitions are achieved realistically during formal patient rehabilitation [41]. Thus, studies isolated to a single rehabilitation setting are unlikely to reveal the neuroplastic implications of a technology like DBWS systems. However, longitudinal studies spanning the continuum of rehabilitation (e.g. inpatient, outpatient, community-based, home-based) may prove helpful to overcoming this barrier.

## Conclusion

This feasibility study supports a benefit of overground therapy with DBWS systems on functional outcomes for persons with NT-SCI undergoing an inpatient rehabilitation course. These findings warrant future prospective, randomized clinical studies of DBWS systems and warrant parallel research to further advance DBWS technology.

## Data Availability

The datasets generated and analyzed during the current study are available from the corresponding author on reasonable request.
